# Near-Infrared Transmittance Spectral Imaging for Nondestructive Measurement of Internal Disorder in Korean Ginseng

**DOI:** 10.3390/s20010273

**Published:** 2020-01-03

**Authors:** Lalit Mohan Kandpal, Jayoung Lee, Hyungjin Bae, Moon S. Kim, Insuck Baek, Byoung-Kwan Cho

**Affiliations:** 1 Department of Biosystems Machinery Engineering, College of Agricultural and Life Science, Chungnam National University, 99 Daehak-ro, Yuseong-gu, Daejeon 341-34, Korea; lalitm85@gmail.com (L.M.K.); snowballgame@naver.com (H.B.); 2 Environmental Microbial and Food Safety Laboratory, Agricultural Research Service, U.S. Department of Agriculture, Powder Mill Rd. Bldg. 303, BARC-East, Beltsville, MD 20705, USA; Moon.kim@ars.usda.gov (M.S.K.); insuck.baek@gmail.com (I.B.)

**Keywords:** near-infrared transmittance imaging, spectral analysis, nondestructive measurement, ginseng, internal disorder, food quality

## Abstract

The grading of ginseng (Panax ginseng) including the evaluation of internal quality attributes is essential in the ginseng industry for quality control. Assessment for inner whitening, a major internal disorder, must be conducted when identifying high quality ginseng. Conventional methods for detecting inner whitening in ginseng root samples use manual inspection, which is time-consuming and inaccurate. This study develops an internal quality measurement technique using near-infrared transmittance spectral imaging to evaluate inner whitening in ginseng samples. Principle component analysis (PCA) was used on ginseng hypercube data to evaluate the developed technique. The transmittance spectra and spectral images of ginseng samples exhibiting inner whitening showed weak intensity characteristics compared to normal ginseng in the region of 900–1050 nm and 1150–1400 nm respectively, owing to the presence of whitish internal tissues that have higher optical density. On the basis of the multivariate analysis method, even a simple waveband ratio image has the great potential to quickly detect inner whitening in ginseng samples, since these ratio images show a significant difference between whitened and non-whitened regions. Therefore, it is possible to develop an efficient and rapid spectral imaging system for the real-time detection of inner whitening in ginseng using minimal spectral wavebands. This novel strategy for the rapid, cost-effective, non-destructive detection of ginseng’s inner quality can be a key component for the automation of ginseng grading.

## 1. Introduction

Ginseng (*Panax ginseng* Meyer) is grown in Korea, northeastern China, and eastern Siberia and is hugely popular for both culinary and medicinal use [1]. Korean ginseng is colloquially referred to as “the king of the herbs” and has been used as a popular herbal medicine for thousands of years [2,3]. Ginseng is commonly used for the treatment of health issues, including ulcers, stress, nervousness, vomiting, tumors, nausea, internal degeneration, inflammation, aging, fatigue, diabetes, and depression [4,5]. Ginseng roots contain a number of active ingredients, including ginsenosides, polysaccharides, phytosterols, peptides, polyacetylenes, fatty acids, and polyacetylenic alcohols [5,6] used for the treatment of the aforementioned health issues. Among them, ginsenosides are known to be the key biologically active components of ginseng. 

Ginseng prices are greatly affected by ginseng quality, which depends upon both external and internal factors, including species, shape, size, species, internal whitening, and pith [6,7]. Dependent on these factors, ginseng samples are graded as 1st, 2nd, or 3rd grade by inspectors (Korea Ginseng & Tobacco Cooperation, 2000) [7]. The presence of inner whitening is one of the most important factors which needs to be assessed for ginseng grading. Whitening levels are dependent on the ginseng growing and processing conditions, which alter the amount of starch found in the root [7], and whitened samples are considered poor quality. 

Today, the internal qualities of ginseng samples are graded visually by skilled inspectors. Samples are illuminated under a light-source, and the inner whitening levels are evaluated by eye [8]. However, visual inspection is not always accurate, and samples are not always graded correctly. Furthermore, this method can be expensive, as it requires the employment of skilled inspectors and experts in every ginseng processing plant. Developing automated, accurate, and non-destructive techniques to replace human inspection and speed up the grading process is a goal for ginseng producers. To date, the quality of ginseng internal tissue has been measured through X-ray and computed tomography (CT). Although these techniques have potential for evaluating ginseng properties [8,9], they were not successfully implemented in the field, due to low accuracy and potentially harmful to human health, so alternative methods are required.

This paper explores the use of a novel technique, using near-infrared transmittance spectral imaging to detect inner whitening in ginseng. Spectral imaging technology, in combination with machine learning algorithms, could provide information to visualize the internal quality of ginseng. Indeed, the technique has already proven successful for the measurement of quality attributes in various agro-food products, including seed, meat, fruit, and vegetable products [10,11]. Spectral imaging is rapid, non-destructive, and accurate. Moreover, both spatial and spectral features are analyzed, which is impossible using regular spectroscopic techniques. 

There are two modes of spectral imaging: reflectance, which is only suitable for the surface identification of objects, and the transmittance mode, which can be used to identify objects’ internal attributes by collecting much inside information. Near-infrared transmittance spectral imaging has previously been used for internal quality detection in various kinds of agro-food products [12,13,14]. However, no study has reported the use of the near-infrared hyperspectral transmittance imaging technique for detecting internal defects in ginseng roots. 

This work uses near-infrared transmittance spectral imaging techniques to detect inner whitening defects in Korean ginseng roots and develops a rapid analysis model for ginseng spectral image data to measure internal whitening levels. We expect that this system will assist the ginseng industry in grading products with a fast, high-throughput detection of internal whitening.

## 2. Materials and Methods

### 2.1. Ginseng Samples

In this work, six-year old ginseng samples were used to study whitening detection using near-infrared transmittance spectral imaging. Ginseng samples of both normal and abnormal materials were obtained from Korea Ginseng Corporation (KGC) 71, Beotkkot-gil, Daedeok-gu, Daejeon, Republic of Korea. The samples of both normal and abnormal materials were investigated and characterized by skilled inspectors from the KGC and included in the experiment. The normal ginseng samples had no whitening in the central region, whereas abnormal samples had distinctive inner whitening. The characterized ginsengs were then analyzed using transmittance spectral imaging measurement. For spectral imaging, 10 normal samples from grade 1 and 10 abnormal samples from grade 3 of different shapes and sizes were used. The shape of the ginseng is more or less cylindrical but sometime protrudes from the sample surface and the thickness of measured ginsengs ranges from 15 to 20 mm. 

**Figure 1:**
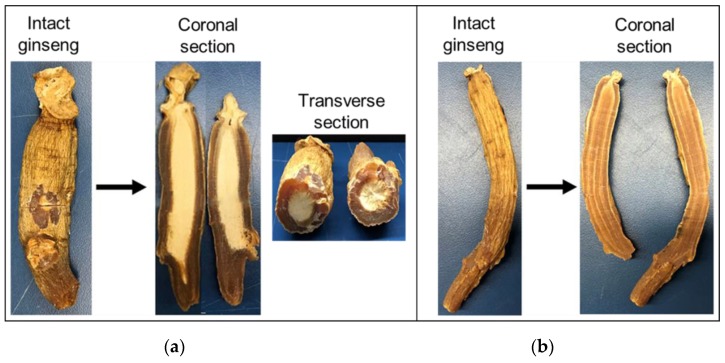
Example of an abnormal and normal ginseng sample. (**a**) Intact and sliced abnormal ginseng with whitening in center; (**b**) intact and sliced normal ginseng without whitening.

Inner whitening was externally undetectable, with both normal and abnormal ginseng samples displaying identical characteristics. The internal profile of abnormal and normal ginseng is shown in Figure 1. 

### 2.2. Near-Infrared Transmittance Spectral Imaging

**Figure 2:**
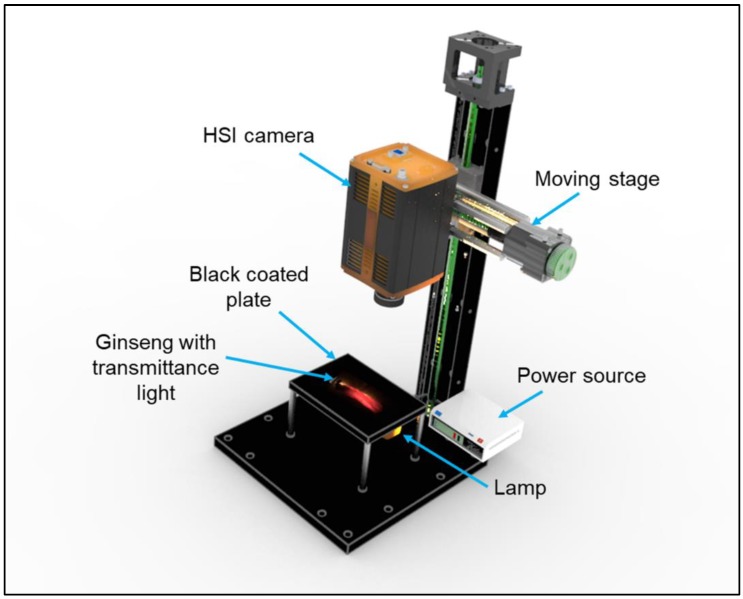
Schematic of near-infrared hyperspectral imaging system used for transmittance imaging of ginseng.

Spectral image measurements of ginseng samples were carried out using a line-scan near-infrared hyperspectral imaging system, operated in transmittance mode (Figure 2). The system comprised a line-scan near-infrared hyperspectral imaging camera (HSI; Pika NIR-320, Resonon Inc., Bozeman, MT, USA), C-mount lens with a focal length of 25 mm f/1.4, and a moving stage. The camera was operated under the following conditions: spectral wavelength range, 900–1700 nm (with 168 spectral bands, 4.9 nm spectral resolution, and 320 × 500 spatial channels), and max frame rate (fps) of 520 Hz. Normally, this system also includes four halogen light sources to illuminate the test samples for reflectance imaging; as this study used transmittance imaging, this work modified the system by replacing the reflectance light source with a transmittance light source. Ginseng samples were illuminated from beneath by a 100 W halogen lamp, placed directly below the sample. To generate uniform lightning conditions for sample illumination, a cylindrical lens was fixed in front of the light source. A computer programmed motorized sample stage was integrated to move the samples towards the camera field of view (FOV). The camera was operated inside a dark chamber to avoid environmental light.

### 2.3. Spectral Image Acquisition

Before HSI images were acquired, the system parameters were adjusted: scanning speed was 2 cm/s, with a total scan of 500 scans/sample. Ginseng samples were placed on the sample holder plate, and a moving stage scanned samples line by line using the HSI system, with a DC motor moving the translation stage towards the camera. The HSI camera was mounted over the stage and began to acquire images as the ginseng sample entered the camera’s FOV. The samples were scanned in the wavelength range of 900–1700 nm. The acquired hyperspectral images were saved in a three-dimensional format containing two spatial dimensions (x and y) and a spectral dimension (λ). Total time of 15 s was required to measure a single ginseng sample with the aforementioned system settings.

### 2.4. Calibraiton of Spectral Images

After hyperspectral image acquisition, a calibration step was performed on raw images to reduce the dark current noise and non-uniform illumination effect from the acquired images. For this purpose, a white and a dark reference image were taken during the measurement. The dark image (0% transmittance) was obtained by turning off the light source and covering the lens, and a bright image (100% transmittance) was obtained with a glass table. Thus, the normalized transmittance value was calculated by applying the following equation:

(1) $$X_{C}\;=\;\frac{T_{ij\;}^{R}\left(\lambda \right)-T_{ij\;}^{D}\left(\lambda \right)}{T_{ij\;}^{B}\left(\lambda \right)-T_{ij\;}^{D}\left(\lambda \right)},$$

where $T_{ij\;}^{R}\left(\lambda \right)$ is the raw transmittance image of ginseng, $T_{ij\;}^{D}\left(\lambda \right)$ is the dark image, $T_{ij\;}^{B}\left(\lambda \right)$ is the white image, and $X_{C}$ is the corrected image. 

After image calibration, an image segmentation method was applied to isolate the background of hyperspectral images from the ginseng samples. For this purpose, a binary mask was created using a threshold value, to allow differentiation of ginseng samples from the background. Later, the background removed hyperspectral images were used for analysis.

### 2.5. Preprocessing of Spectral Images

Preprocessing is an important step in the analysis of hyperspectral images and can be used to remove unwanted variation in the data, while retaining as much useful information as possible. Three well-known preprocessing methods (mean, max, and range normalization) were tested for preprocessing the ginseng hyperspectral image data. Normalization involves the fitting of data within unity by correcting the mean, maximum, and range values, so all data values take on a value between 0 and 1 [15,16]. After testing all three preprocessing methods, range normalization was found to be the most appropriate method for removing noise from the hyperspectral image data. 

### 2.6. PCA-Based Optimal Wavelength Selection for Detecting Whitening 

**Figure 3:**
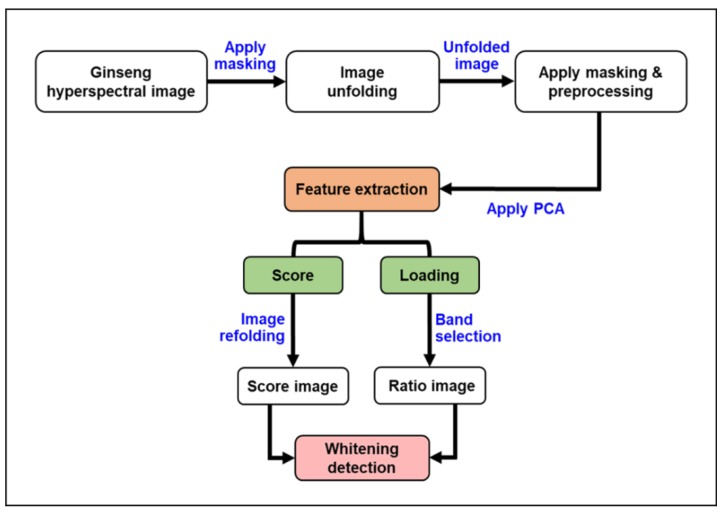
An illustration of hyperspectral image processing sequences used for whitening detection in ginseng.

Principal component analysis (PCA) was used on the preprocessed images (Figure 3). PCA is an analytical method, used for high dimensionality data decomposition, feature selection, and feature visualization in hyperspectral data. It transforms high dimensional data into lower dimension data (also called principle components, PCs), by linear transformation. Graphics displaying PC scores visualize the features of multivariate data into smaller dimensions and illustrate differences and similarities among the samples. Factor loading is used to quantify how much each original variable contributes to a given PC [17].

To apply PCA to 3D hyperspectral ginseng matrices, it is necessary to unfold the hypercube into a 2D matrix, in which each column represents all the pixels from a given spectral band in the original image cube, and each row represents the spectrum of a single pixel [18,19]. Furthermore, the PCA was done by applying the following equation:

(2) $$X_{C}\;=\;T_{ij}P_{ij}^{T}+\;E_{ij},$$

where $X_{C}$ is the unfolded 2D matrix, $T_{ij}$ is the score matrix, $P_{ij}^{T}$ is the loading matrix, and $E_{ij}$ is the error matrix.

The calculated scores matrix was re-folded, displaying the results in a score image that illustrates the distribution of the components in ginseng samples. The loadings obtained from the PCA were used to select optimal wavebands to detect inner whitening in ginseng. These optimal wavelengths, identified from the PC loadings, can be used for multispectral detection of whitening in abnormal ginseng samples. PCA and image processing techniques were carried out in MATLAB (2016a, MathWorks Inc., Natick, MA, USA).

## 3. Results and Discussion

### 3.1. Spectral Characterstics of Ginseng

**Figure 4:**
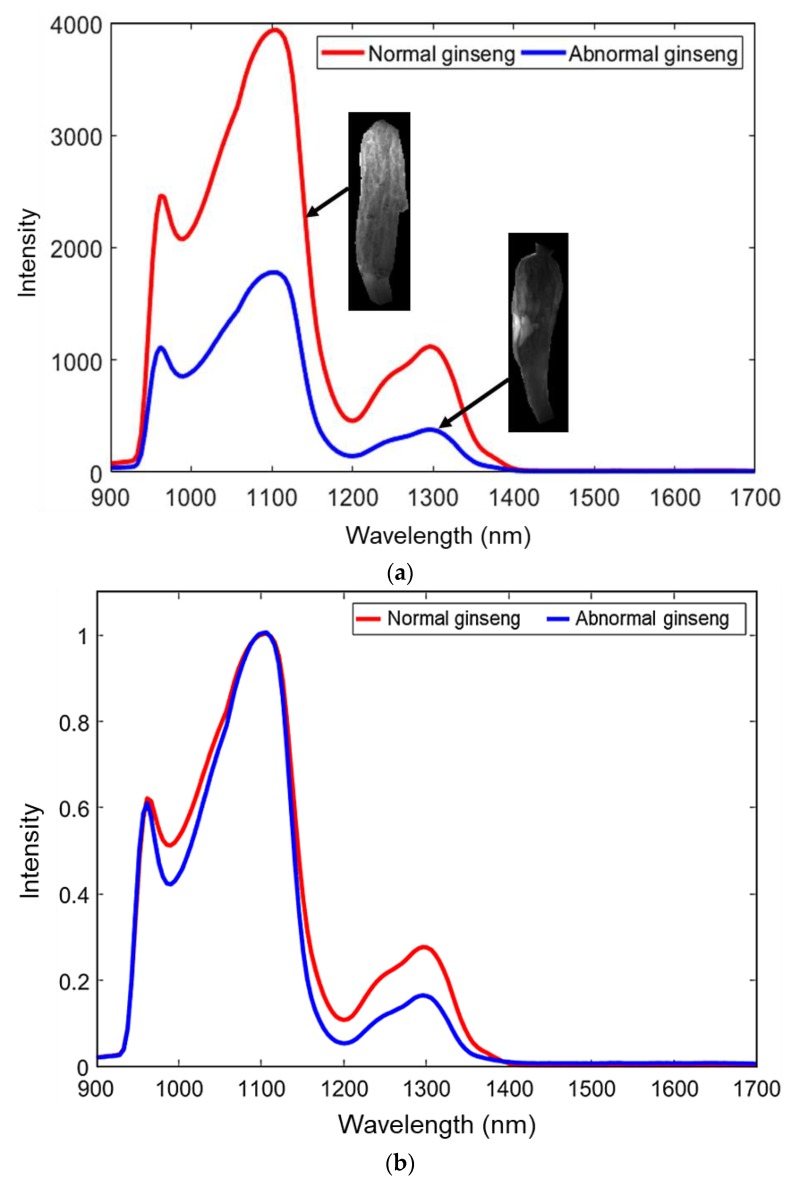
(**a**) Raw spectrum and corresponding hyperspectral images of normal and abnormal ginseng samples; (**b**) normalized spectrum of normal and abnormal ginseng samples.

Figure 4a,b show the raw and normalized spectrum of normal and abnormal ginseng samples with corresponding hyperspectral images. The raw hyperspectral images showed that whitened ginseng exhibits a darker region in center of the transmittance image, with differing intensity (Figure 4a ginseng spectrum with corresponding hyperspectral images) [20]. The occurrence of darker regions in the center is the result of higher optical density in the whitened material. In Figure 4a,b the spectrum of abnormal ginseng samples reflected lower transmittance intensity characteristics than that of the normal ginseng, especially in the region of 900–1050 nm and 1150–1400 nm, respectively. This is due to the inner, whitened region of abnormal ginseng reflecting higher amounts of light in this spectral region.

### 3.2. Whitening Detection Based on PCA and Two-Band (Ratio) Images

**Figure 5:**
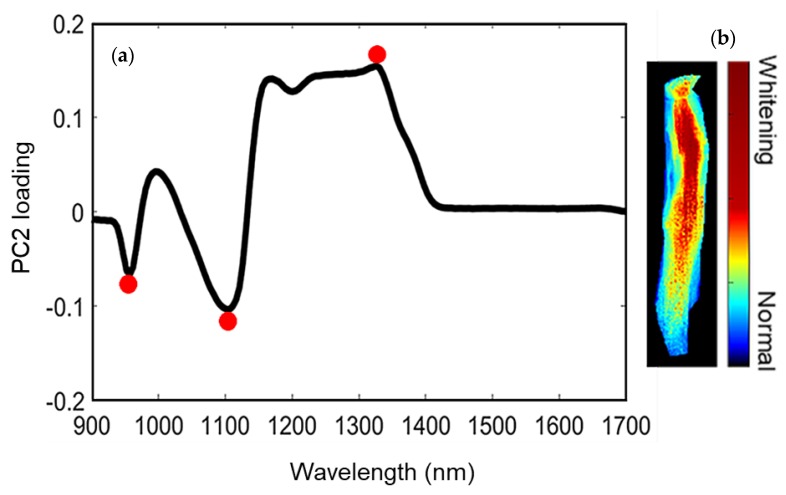
(**a**) Second principal component (PC2) loading of ginseng showing the characteristic wavebands; (**b**) PC2 score image of ginseng based on the characteristic wavebands.

The ginseng hyperspectral data was analyzed using PCA, in combination with the image processing technique. In this study, PC-2 loading (Figure 5a) was chosen for ginseng characterization, as PC2 made the greatest contribution to detecting inner whitening (Figure 5b, PC-2 score image). Overall, the first three PC loadings accounted for nearly 80% of the total sample variance; subsequent PCs were unimportant and probably reflected noise in the data. Overall, PC-2 preserved relatively complete information regarding the internal quality of ginseng, and PC-2 loading showed characteristic bands at 950, 1110, and 1326 nm, which are associated with the ginsenoside compound ($C_{42}H_{72}O_{14}$) of the ginseng sample [21]. Specifically, the band at 1326 and 1110 nm corresponds to the C-H stretching vibrations of ginsenoside [21], and the band around 950 nm corresponds to the second overtone of the O-H stretching vibration [22] of the ginsenoside compound. 

In the PC-2 score image (Figure 5b), the whitened region in the center of the sample appeared bright, whereas in the PC-1 and PC-3 images (not shown), this region was dark. As PC-2 was able to discriminate the whitened region from the surrounding healthy tissue, the PC-2 loadings can be the feasible wavebands for detecting whitening [23]. On the basis of the above observations, the two most differentiating wavebands (950 and 1326 nm) from the PC-2 loading were used to carry out image-based classification of whitened regions. This approach may provide information for the development of low-cost multispectral imaging techniques to measure the internal quality of ginseng. In addition, the one-way analysis of variance (ANOVA) test was also conducted in R software (version 3.6.1) for each waveband considering abnormal and normal ginseng. The ANOVA test showed that the wavelength region 950 and 1326 nm have significant differences (better classification) between abnormal and normal ginseng, as the larger *F*-value of 12 and lower *p*-value of 0.002 (*p* < 0.05) is in this wavelength region. The larger *F*-value indicates a more statistically significant mean separation between two groups [24].

**Figure 6:**
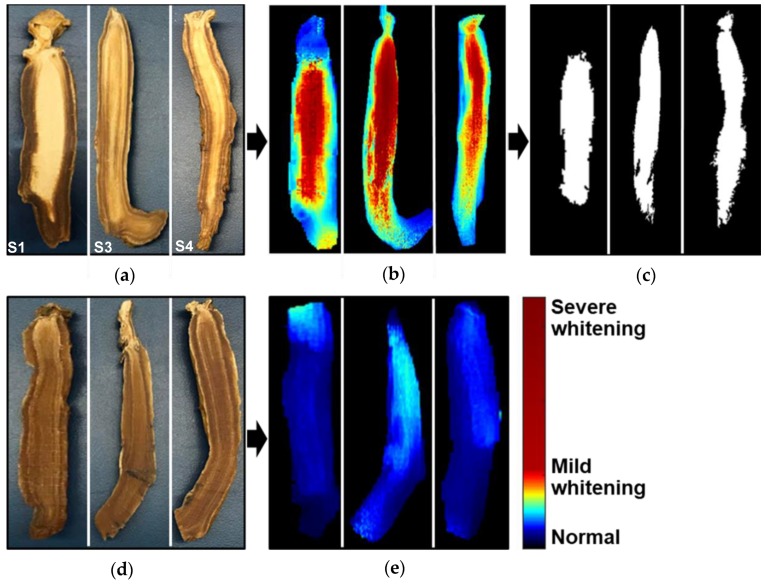
(**a**) RGB images of sliced abnormal ginseng samples (S1: 48.18% whitening, S3: 53.14% whitening, and S4: 37.66% whitening); (**b**) ratio image visualizing the whitening region (red pixels) in abnormal ginseng samples; (**c**) binary image of detected whitening region in abnormal ginseng samples; (**d**) RGB images of sliced normal ginseng samples. (**e**) Ratio images of normal ginseng samples. Coloration map has been added to the ginseng and the whitened region, to enhance visual discrimination.

Later, by applying the optimal wavebands identified through PCA, we saw that certain image ratios (Figure 6b) made whitened regions more apparent, whereas whitening was absent in the normal samples (Figure 6e). In contrast to the normal ginseng samples, the abnormal ginseng showed more warm coloration (yellow-red) in the center, where normal ginseng showed more cool coloration (green-blue). The PC-2 score images (Figure 5b) showed a similar coloration map for discriminating between the normal ginseng and abnormal ginseng. 

After the hyperspectral measurement of ginseng samples, some normal and abnormal ginseng samples were halved to confirm the presence of whitening (Figure 6a,d). The color images of abnormal samples (S1, S3, and S4) showed a central whitening amount similar to the detected percentage of whitened pixels in the binary image (Figure 6a,c). This demonstrates a good correlation between the real images and the pixels detected nondestructively in the hyperspectral images. Normal ginseng slices showed no sign of whitening in the central region (Figure 6b).

### 3.3. Identficaion of Whitening Region on the Basis of Binary Imaging

**Figure 7:**
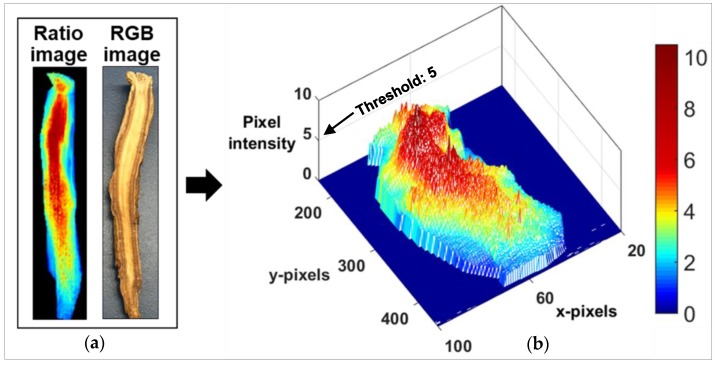
(**a**) Ratio and RGB images of an abnormal ginseng sample used for mash plot development. (**b**) 3D mash plot showing ginseng pixel intensity; the x and y-axis represent the pixel identities, whereas, the z-axis represents the pixel intensity. The red region in the plot belongs to the whitening region. Threshold value of 5 was used to detect the whitening pixels, as the larger proportion of whitening pixels lies above this threshold value.

The binary images (Figure 6c) were used to assess pixilation in the whitened region. To generate binary images from the ratio images, image segmentation was used, based on a threshold value. To determine a suitable threshold value, a 3D mash plot was produced, reflecting the ginseng image pixel intensity (Figure 7). In this plot, the majority of the whitened pixel intensity (red-region) lies over 5, so a threshold value of 5 was used to segment the whitened region from the remainder of the ginseng sample. All pixels below this threshold were subsequently classified as 0 (black); all pixels above this threshold were classified as 1 (white). The following equation was used to detect the whitening region of the abnormal ginseng.

(3) $$g\left(x,y\right)\;=\{\begin{array}{c} 1\\ 0 \end{array}\begin{array}{c} if\left(x,y\right)\geq T\\ otherwise \end{array},$$

where g(x,y) represents whitening image pixel at (x,y), f(x,y) represents the original ginseng image pixel at (x,y), and *T* represents the threshold value.

This study tested several threshold values, but some pixels were unidentifiable when ginseng had little or no whitening. The threshold value was best at identifying pixels when there was little or no whitening. After the threshold value was applied, the resultant binary images (Figure 6c) showed that pixels above the threshold value corresponded to whitening, whereas pixels below the threshold value corresponds to healthy ginseng. The binary images isolated whitening from surrounding healthy regions. Further percentage whitening in ginseng samples was also calculated, using the following equation:

(4) $$T_{p}=\;W_{p}\;+\;H_{p},$$

(5) $$P_{wp}\;=\frac{W_{p}}{T_{p}}\;\times \;100,$$

where $T_{p}$ is the total number of ginseng pixels, $W_{p}$ is the total number of whitening pixels in ginseng, $H_{p}$ is the total number of normal pixels in ginseng, $P_{wp}$ is the percentage of whitening pixels in the ginseng [25]. 

**Figure 8:**
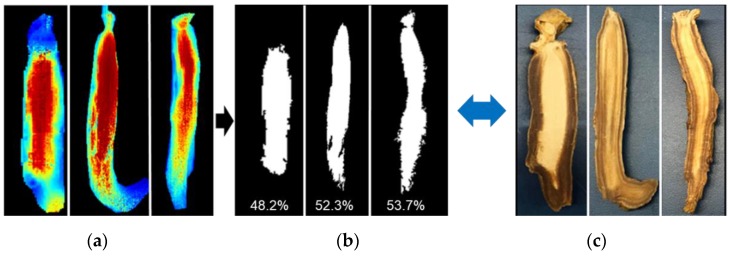
(**a**) Nondestructive ratio image visualizing the whitening region (red pixels) in abnormal ginseng samples; (**b**) binary image of detected whitening region; (**c**) color images of sliced abnormal ginseng samples for comparison.

By applying Equations (4) and (5), it was found that the percent range of heavy whitening ranged between 48–54% in the measured abnormal ginsengs. The calculated number of whitened pixels was based on the size/area of whitening in the samples; the extent of the whitened region was confirmed by comparing the generated hyperspectral images with color images of abnormal ginseng, showing a good match between the images as shown is Figure 8. In addition, we have also applied an outlier detection method using the 3σ rule, however, no outliers were observed in the calculated pixels.

## 4. Conclusions

Near-infrared transmittance spectral imaging was successfully used to detect inner whitening in ginseng samples. The transmittance intensity of abnormal ginseng was generally lower than that of normal ginseng samples. Firstly, PCA was conducted to identify the most effective wavebands pairs for differentiating whitened from healthy regions. The PC-2 score images showed the best discrimination between normal and whitened regions, so the effective wavebands from PC-2 loadings were used to generate a two-band ratio image. This ratio image showed good visualization of the whitened region in abnormal ginseng samples. Thus, the study shows that is it possible to develop an economic spectral imaging system to evaluate internal ginseng properties, using only two spectral bands. Our results suggest that near-infrared transmittance spectral imaging is a suitable and rapid method for grading ginseng and could be an alternative to using skilled inspectors. Considering the time saved with this method, it is much faster compared with the current manual grading system (based on human visual inspection). Manual grading is time-consuming, as it takes one or two days to detect the inside properties of a single ginseng lot. By adopting this system in a ginseng plant, it could change the grading system from days to hours, due to the automated process and analysis. In the future, spectral imaging techniques using this approach could be used to measure the internal quality of ginseng and could be developed into a key automated method for ginseng producers.
